# The open health-promoting activities programme: redefining health promotion and family dynamics by engaging parents in socioeconomically deprived Swedish communities

**DOI:** 10.1186/s12889-025-21799-0

**Published:** 2025-02-12

**Authors:** Lisette Farias, Mai-Lis Hellenius, Johanna Gringmann, Gisela Nyberg, Susanne Andermo

**Affiliations:** 1 Department of Neurobiology, Care Sciences and Society, Division Of Nursing, Karolinska Institutet, Huddinge, 141 83 Sweden; 2 Department of Neurobiology, Care Sciences and Society, Division of Occupational Therapy, Karolinska Institutet, Huddinge, 141 83 Sweden; 3 Department of Medicine, Karolinska Institutet, Solna, 171 77 Sweden; 4 The Swedish Gymnastics Federation, Stockholm, 100 61 Sweden; 5 Department of Physical Activity and Health, The Swedish School of Sport and Health Sciences, Lidingövägen 1, Stockholm, 114 33 Sweden; 6 Department of Global Public Health, Karolinska Institutet, Stockholm, 171 77 Sweden

**Keywords:** Family intervention, Disadvantaged populations, Children, Play

## Abstract

**Background:**

Current evidence suggests that even in high-income countries such as Sweden, there are socioeconomic differences in children’s participation in physical activity. While family-based programmes appear promising to encourage physical activity, there is a lack of knowledge on how to engage families in such programmes, particularly in socioeconomically disadvantaged areas. The Open Health-Promoting Activities programme was launched to promote physical activity outdoors and health equity for children and their families in these areas. This study aims to explore parents’ experiences with the Open Health-Promoting Activities programme in socioeconomically disadvantaged areas, focusing on family engagement in physical activity and perceived changes in family dynamics.

**Methods:**

A qualitative design with an ethnographic approach was employed. In line with an ethnographic approach, the research team conducted 15 participant observations of the programme sessions on Saturdays during Spring 2022. Field notes were compiled during the observations, which provided contextual information for individual interviews with 12 programme participants. These interviews were conducted after the researchers attended the programme. The participants were adults/parents who participated in the programme with one or more of their children. An inductive reflexive thematic analysis was used to analyse the field notes and interviews.

**Results:**

The analysis identified three main themes: (1) prioritising children’s equal engagement in physical activity, (2) helping parents promote children’s healthy lifestyles, and (3) improving family dynamics through engagement in physical activity. Each theme captures an aspect of the programme that parents perceived as essential to facilitating their family’s engagement in the programme. All the themes are interconnected and form the basis for improving family dynamics.

**Conclusion:**

To develop tailored family-based programmes in socioeconomically deprived communities, it is crucial to understand parents’ experiences and perceptions of aspects that facilitate their children’s engagement in physical activity. The findings suggest that emphasising equal opportunities, a safe space approach and participation are essential for increasing family engagement in physical activity. These elements also supported increasing parents’ awareness of their children’s need to be active and have fun together.

**Supplementary Information:**

The online version contains supplementary material available at 10.1186/s12889-025-21799-0.

## Introduction

Despite the proven benefits of physical activity (PA) for the physical, social, and mental health of children and adolescents [1–3], most children and adolescents do not meet PA guidelines [4, 5]. Current evidence in high-income countries indicates socioeconomic disparities in PA, such as sports participation, among children [6]. In high-income countries, such as Sweden, children from high socioeconomic status (SES) backgrounds are more likely to participate in organised sports than those from low SES backgrounds [6]. Low parental SES indicates that children may have more difficulties accessing organised sports [7, 8]. These difficulties are associated with lack of access, lack of support, safety concerns, financial costs [9], aesthetics of the environment (i.e., problems with cleanliness and maintenance), and parents’ lack of time to take them [9]. Given the health benefits of PA, it is essential to promote an equity focus on programmes to increase access and opportunities to be physically active, particularly in socioeconomically disadvantaged areas [6].

Children spend considerable time in the care of their parents, suggesting that parents are not only role models but also ‘gatekeepers’ to children’s lifestyle habits, including PA participation [10]. In addition to parental SES, parents’ involvement in sports can significantly predict children’s PA participation [11–13]. Parents and families are vital sources of influence and promotion of children’s PA, as they can encourage, model, and engage in PA with their children [14, 15]. Although family-based programmes seem promising to enable children’s PA, there is a lack of knowledge on how to facilitate the recruitment and retention of families in such programmes, specifically in socioeconomically disadvantaged areas [16]. These challenges are particularly significant when lower PA levels are consistently reported among children in low SES contexts [17, 18].

To improve the health equity of families in disadvantaged areas in Sweden, a non-profit organisation, ‘A Healthy Generation,’ launched a school-based programme in 2011. During the COVID-19 pandemic, a global decline in PA among children has been reported [19, 20]. Nevertheless, in countries like Sweden, where children could engage in outdoor play during the pandemic, children aged 11–13 generally maintained their PA levels [21]. However, opportunities for outdoor play varied among groups in Sweden, indicating that children from socioeconomically disadvantaged areas engaged in less PA [22] and that pre-existing social inequalities in PA increased during the pandemic [22, 23]. Consequently, the non-profit organisation A Healthy Generation created the programme ‘Open Health-Promoting Activities’ to promote PA outdoors among families in socioeconomically disadvantaged areas of Sweden.

Research on the experiences of families participating in PA programmes within socioeconomically disadvantaged areas is limited [16]. Interventions to increase the level of PA in families with low SES are needed to reduce social inequalities and improve the well-being of the Swedish population [22]. Research and knowledge about the effects of regular PA and other healthy behaviours are increasing [24], but its effectiveness is limited without adequate implementation. In Sweden, health inequalities are growing rapidly [25], and there is an urgent need to find new and more effective implementation strategies to close these health gaps. To support the development and implementation of family-based PA interventions tailored to the needs of families with low SES, an understanding of their experiences is crucial. This study aimed to explore parents’ experiences with the Open Health-Promoting Activity programme in socioeconomically disadvantaged areas, focusing on family engagement in PA and perceived changes in family dynamics.

## Methods

### Study design

**Fig. 1:**
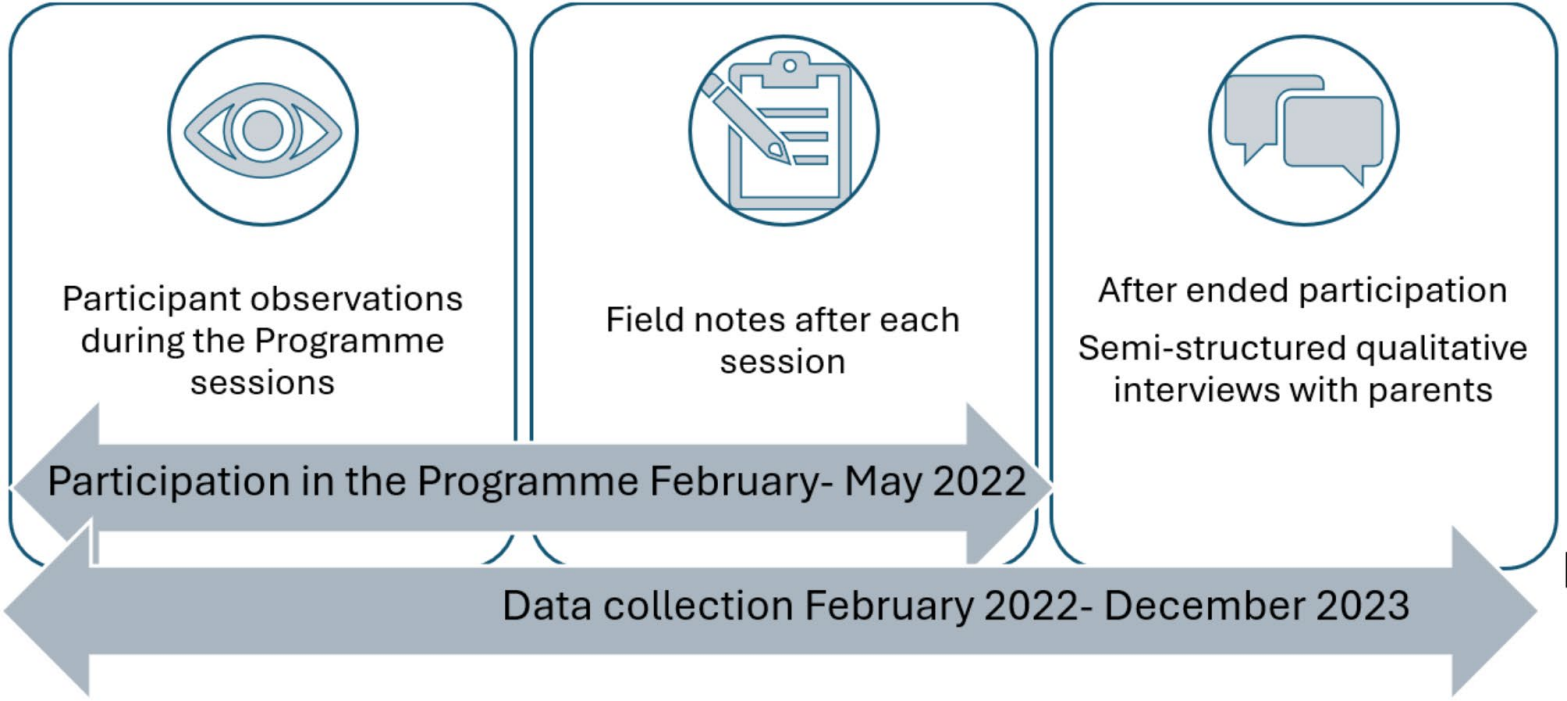
Overview of study design and methods

The study employed a qualitative design and an ethnographic approach [26, 27] to obtain detailed and comprehensive insights into participants’ experiences and engagement in the programme. The researchers conducted participant observations and took notes during the activity sessions to elicit an ethnographic approach. Following researchers’ participation in the sessions, semi-structured qualitative interviews with parents who had participated in the Open Health-Promoting Activities were conducted [27] (See methods section for a description of the interviews). Data collection took place from February 2022 to December 2023. Multiple methods and prolonged researcher engagement have been employed to obtain valuable and complementary insights, which may be difficult to access by relying on a single data collection method [28]. The choice of multiple methods is consistent with the social constructivist epistemological underpinning of the study, which recognises that participants’ perspectives are constructed along with everyday interpersonal interactions within specific cultural contexts [29]. For example, observations enhanced the data collection and analysis with broad and compound empirical material documented in the field notes to examine participants’ situated interactions anchored in their social living conditions [30, 31]. The study is reported according to COREQ guidelines [32]. See Fig. 1 for an overview of Study design and methods.

### The open health-promoting activities programme

The Open Health-Promoting programme (abbreviated to Open Activities) was created by the non-profit organisation ‘A Healthy Generation’ to respond to the need for outdoor activities for families with children living in socioeconomically disadvantaged areas during and after COVID-19. Unlike the A Healthy Generation school-based programme, which conducts school activities and requires families to register to participate, Open Activities is an outdoor, drop-in family programme held in selected socioeconomically disadvantaged areas. The National Board of Health and Social Welfare has developed a set of algorithms for distinguishing between ‘privileged’ and ‘disadvantaged’ (or ‘poor’) areas [33]. This established information is publicly available for municipalities and programmes such as the Open Health-Promoting Activities programme.

The Open Activities programme is open to families with children, especially those between 6 and 12 years old. At least one parent is required to participate. The parents are self-identified and often adults living in the same household as their children. However, other family members may also participate, such as children accompanied by their uncle, grandparents, or parents of another child if their parents cannot attend a particular session.

Initiated in September 2021, the Open Activities programme was implemented in 16 municipalities in Sweden. The programme has been carried out in several socioeconomically disadvantaged areas in some municipalities. The Open Activities programme is held every weekend for one hour and is led by a health coordinator either in a central park or a football field. Information about the Open Activities programme is disseminated through various channels, including word-of-mouth, the distribution of flyers in schools, and the efforts of health coordinators, who engage with local communities in the surrounding area 30 min before the activities start and invite families who are in public spaces, such as parks and playgrounds.

The initial 45 minutes of the programme are dedicated to activities centred on games and sports, which are conducted in a manner that involves parents and children. Health coordinators plan a series of games and sporting activities before the sessions. They ensure that the necessary equipment, such as coloured vests, cones, and balls, is available to conduct the activities and mark the boundaries of the playing areas and groups. The games are frequently adaptations of traditional games such as ‘Simon Says’, ‘Tag’, ‘Statues’, ’Duck, Duck, Goose’, and ‘Captain, Captain’, in which children and families engage in friendly competition within intergenerational mixed-gender groups. The activities are designed to engage children and adults in pursuing a mutual objective, whether chasing others or collecting items. As a drop-in programme, the activities are adapted to the group size, which can vary from session to session. The average group size observed by the researchers was between 8 and 15 children and 6–8 adults. The remaining 15 min are dedicated to answering quizzes about healthy habits and offering fruit to all families. Attendance in the programme is free of cost.

The Open Activities programme remains in operation and is overseen by the A Healthy Generation organisation, which recruits and trains the health coordinators responsible for its implementation. The health coordinators are responsible for developing games and activities conducted with families. The organisation and the staff who organise the activities provide resources to the health coordinators to facilitate implementing these activities. The research team responsible for evaluating the Open Activities Programme is external to the organisation and the health coordinators.

### Recruitment and participants

Parents (adults) of both genders who had participated in the Open Activities programme with at least one of their children from 2022 to 2023 were recruited for semi-structured qualitative interviews. Parents were recruited during the programme activities, where they were provided with information about the study and had the opportunity to become acquainted with the researchers. During the participant observations, LF, JG, and SA introduced themselves and provided written information about the study to all participants. The inclusion criteria were participating in the programme with one or more of their children, 18 years old or older, and willing to share their experiences about the programme in Swedish or English. The participants provided their contact information directly to the researchers or a health coordinator. With this information, JG and LF contacted participants and booked an interview with each participant at a time and place of convenience. The health coordinators also reminded potential participants about the study during data collection.

A total of 12 participants were recruited for the study (9 females and 3 males). Except for one participant, most participants had a foreign background. The children of the participants who participated in the program were between 9 and 12 years old. Most participants were university students or had obtained a preschool teacher assistant or assistant nurse degree through vocational tracks in high school or municipal adult education (Komvux), which caters to adults who lack an education from compulsory or upper-secondary school. Notably, both university-level and municipal adult education in Sweden is tuition-free. Additionally, students can apply for financial assistance through various grants and loans offered by the Swedish Board of Student Finance. These resources can be utilised to cover essential living expenses during their studies. The participants’ characteristics are shown in Table 1.

**Table 1:** Participants’ characteristics

Gender	Marital Status	Age	Number of children	Occupation
Female	Married	43	4	Preschool teacher assistant
Female	Married	37	4	Student, Swedish as a second language (high school or municipal adult education)
Male	Married	44	3	Research Specialist
Female	Married	48	3	Preschool teacher assistant
Female	Married	33	2	Parental leave. Preschool teacher assistant
Female	Married	48	3	Preschool teacher assistant
Male	Married	43	2	Export operations worker
Female	Married	33	3	Part-time worker in home healthcare and university student
Female	Married	39	3	Part-time worker as a doula and university student
Female	Married	49	3	University student full-time
Female	Married	48	4	Assistant nurse
Male	Divorced	48	3	Warehouse worker

## Data generation

### Participant observations and field notes

Participant observations were conducted during spring 2022 by LF, JG, and SA. A total of 15 observations were carried out for 1–1.5 h each Saturday during the Open Activities, except for one time when the activities were suspended. Moderate to active participation [26] was used, meaning that the researcher did not take initiatives directed at the families participating in the activities but responded to questions and interacted with participants during the activities by playing the same games. The observations provided an opportunity to obtain a complete picture of the Open Activities in their cultural and socioeconomic context. For example, it allowed researchers to gain insights into the role of the health coordinator, how the activities were conducted, how families engaged, and how the weather affected the activities. After the observations, field notes were taken using a grid elaborated for this study and based on ethnographic principles [25] to document the sessions. This documentation included the date, location, number of participants, physical environment, social interactions, type of activities, and direct quotes from participants. This documentation also included the researcher’s reflections and questions that emerged from the observations.

### Semi-structured qualitative interviews

LF and JG interviewed the participants. LF is a female researcher at a Swedish university with experience and training in conducting qualitative research with participants from different cultural and socioeconomic backgrounds. JG is a female master’s student in sports science with previous experience as a health coordinator in the A Healthy Generation programme in a geographical area different from the one where the study was conducted. The initial five interviews were conducted jointly by LF and JG, whereas the subsequent interviews were conducted solely by LF. The interviews were conducted following a semi-structured interview guide developed by the research team (please see the interview guide supplementary file). The interview guide comprised ten open-ended questions to elicit comprehensive responses during the interviews. The interviews included questions about how the parents were introduced to the Open Activities, their views on the types of activities, accessibility, and aspects that contributed to or hindered their future participation.

The interviews were conducted in various formats following the participants’ requests. Six participants agreed to a face-to-face interview immediately after attending the activities in a community centre near the park where the activities took place. One of these participants requested that a friend be present during the interview because of difficulties communicating in Swedish. Five participants agreed to a mobile phone interview due to time and work constraints. In contrast, one opted for an email response to the semi-structured interview guide to overcome language barriers. LF and JG provided the option of conducting interviews in English or Swedish. Eleven participants preferred to conduct the interviews in Swedish, and only one chose to be interviewed in English. A total of 12 individual interviews were carried out with parents. All interviews, which ranged from 25 to 46 min, were audio recorded and transcribed by LF or JG.

## Ethical considerations

Ethical approval was received from the Regional Ethical Review Board in Stockholm (Dr no: (2022-02643-02). All participants in the Open Activities programme were informed about the study and the right to withdraw at any time during the study period, both orally and in writing. Consent was gathered s from all families participating in the Open Activities before observation. All parents provided their written consent before the individual interviews. The collected data, including audio files, transcriptions, and field notes, were securely stored and managed.

## Data analysis process

LF, JG, and SA were primarily responsible for the data analysis. M-L H and GN were involved in the final stages of data analysis, which entailed the review of themes and preliminary results. M-L H, SA, and GN previously participated in the evaluation of the A Health Generation school-based programme and provided a comprehensive understanding of the context of the study. An inductive reflexive thematic analysis (TA) [34] was used to identify and analyse themes that capture essential features in the data concerning the study’s aim. Braun and Clarke’s [34] six-phase process was used as a framework to conduct the analysis, involving constant movement back and forth through the phases. This movement led to new interpretations of the data, which required further revisions of the whole dataset, the coded data extracts, and developed themes [35]. To ensure trustworthiness, Braum and Clare’s [36] questions for evaluating TA were used to assess the quality of the study. These questions serve as a guideline for determining aspects such as the motivation for using the TA, the type of TA employed (reflexive), and the fit between the epistemological underpinning of the study and the methods used for data collection and the TA.

Familiarisation with the data started by transcribing, rereading the entire dataset, and checking all the transcripts against the audio files for accuracy. The transcripts were read several times to understand the data as a whole and to note initial trends in the data and potentially interesting extracts from the transcripts. The initial coding of the entire dataset was facilitated by using the Atlas.ti 23 programme, providing equal consideration of all transcripts. The programme facilitated the coding by providing a more accessible overview of the data and more than one code for some parts of the data. Some codes were refined in later iterations of the coding, whereas others were prevalent throughout the entire data set and guided the development of themes.

All the coded data were then organised into preliminary themes, which were reviewed and refined. Some themes were combined and reorganised at this stage based on shared meanings. This process generated themes and subthemes by aggregating the codes that shared a similar underlying concept or meaning across the dataset. These preliminary themes were checked against the coded extracts and the entire dataset, including field notes of observations, to produce a thematic map of the analysis. Several codes were mapped via Atlas.ti 23 to facilitate the visualisation and reorganisation of the codes and themes. The authors refined the themes through discussion, generating clear definitions and names for each theme. After the final themes were drafted, quotations were selected to produce the final findings section.

## Findings

**Fig. 2:**
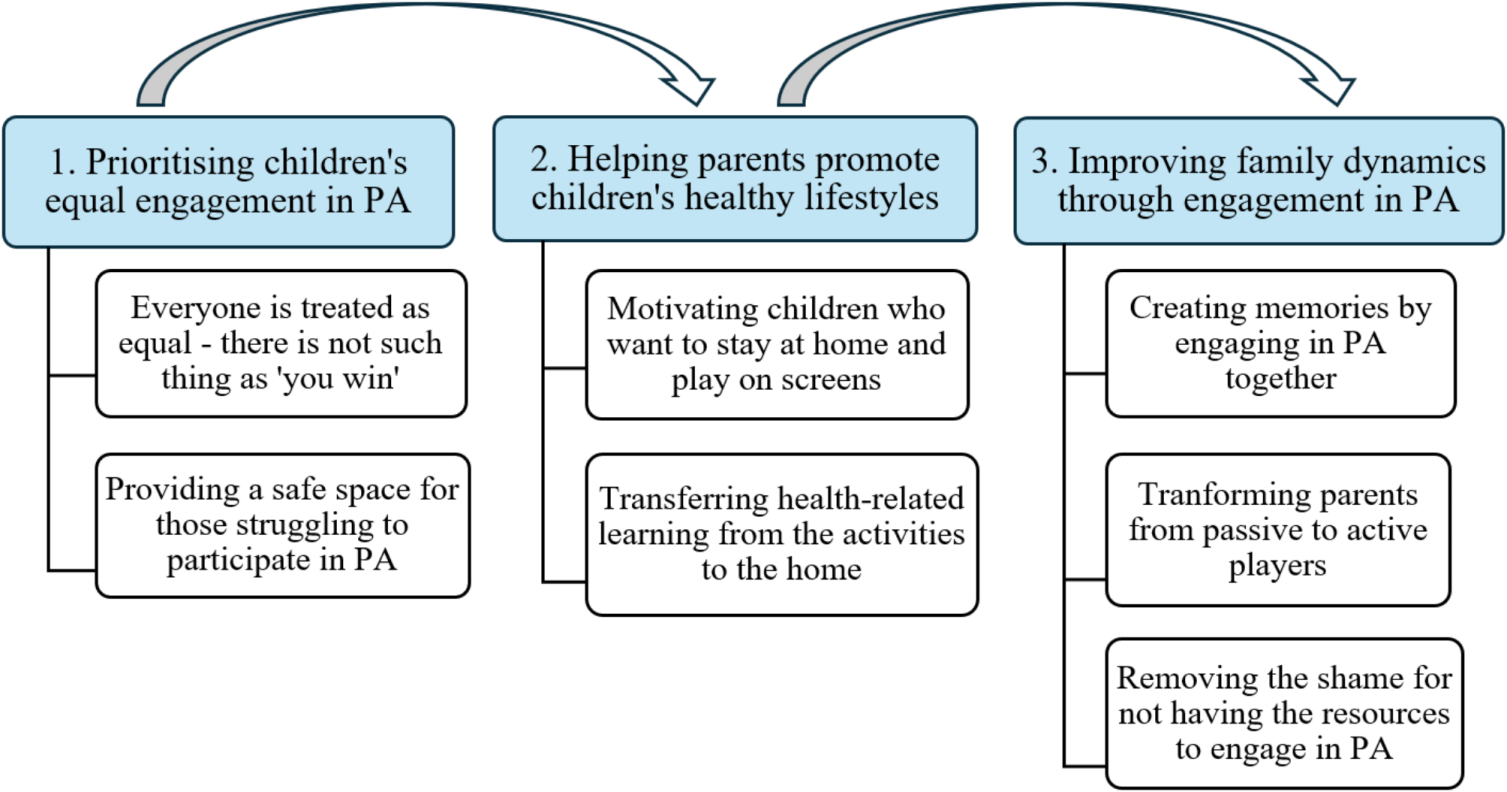
Overview of themes and subthemes

The analysis generated three themes: (1) prioritising children’s equal engagement in PA, (2) helping parents promote children’s healthy lifestyles, and (3) improving family dynamics through engagement in PA. Figure 2 shows the thematic map of the main themes and corresponding subthemes. The main themes focus on parents’ experiences of the provision of the Open Activities programme and how this relates to their family engagement in PA and perceived changes in family dynamics. Each theme captures an aspect of the programme delivery that parents perceived as essential to facilitating their family’s engagement in PA. All the themes are interconnected, forming the basis for improving family dynamics. For example, treating children as equals and creating a safe space is a foundation for motivating them to engage in PA instead of staying at home and playing video games. This safe space, in turn, allows families to create meaningful memories in which parents are now active players and role models.

## Theme 1: prioritising children’s equal engagement in physical activity

### Everyone is treated as equal - there is no such thing as ‘you win’

As the participants repeatedly mentioned, the opportunity to have activities that treated all the children as equals was something remarkable in the delivery of the programme activities. This aspect was associated with age-appropriate activities and accommodating children’s developing skills and understanding. The parents mentioned, “You noticed that all activities were suitable for all children” (interviewee 4) and that health coordinators “paid attention to the children and the children’s knowledge of the activities” (interviewee 6). For instance, the health coordinators ensure they memorise the names of all the children and parents and often ask for their opinions and impressions of the activities and games. The activities were also described as encouraging all children to participate on equal terms yet acknowledging their differences and supporting those who needed support. On several occasions, when children and parents had difficulties understanding health coordinators in Swedish, the coordinators used nonverbal communication to explain the rules of the games, such as body language or walking with the participants to signal when to catch, throw, or pick up a ball. The parents felt that the most critical aspects of the activities were avoiding competition, accepting differences, and facilitating cooperation and friendship between children. One parent commented: “The most important thing is that there is no such thing as ‘you win’, always go with the same treatment plan, everyone is equal even if different, everyone has the Convention of the same rights on the Rights of the Child” (interviewee 4).

The parents identified equal treatment as crucial for maintaining motivation and continuing to participate with their children in the activities. This approach helped them reduce their concerns about their children feeling excluded because of differences in skills or resources.

You have your child on a football team, for example. But he’s not that good. You can’t play a game, or you don’t have the money? Then I get a bit worried that it’s unfair, you know? But with Open Activities, it’s like that: it doesn’t matter how bad or good you are, you’re still in the same group (interview person 8).

### Providing a safe space for those struggling to participate in PA

Parents of children who had difficulties engaging in social activities emphasised that these activities provided a safe space for their children to meet new peers. This safe space was particularly noticed by parents, who described their children as introverted.

She is so shy. I notice that when she plays there, she doesn’t need me to be there. And she talks about these activities […] When we were at the Open Activities, it automatically showed that she was in and is part of those groups (interviewee 6).

During the observations, a parent mentioned that the activities provided a safe space for her child with attention deficit hyperactivity disorder (ADHD) to have fun without revealing his concentration difficulties. Parents of children who usually find it challenging to join new groups noted that by participating in the activities, the children could try new activities and test their abilities in a supportive environment. A parent commented that participation in the activities boosted her child’s confidence and encouraged her to try activities she had never done before.

It meant for her that she got to know her body better. And say that she can and has fun, without noticing that that she does certain things that she hasn’t done before. I also see this development [in her] that you do not always know that you develop in this, to lose to win, to be in the middle, to cheat (interviewee 10).

Children’s feeling of safety supported their engagement in peer activities. Several parents also observed their children testing how to collaborate with others. “I think it’s great fun, and the children also think so; they cooperated with the other children through playing” (Interviewee 2). A parent explained how the activities helped her child become more social by assisting other children who had difficulties getting to know new peers or following the activities.

He, who is 10 years old, usually talks about the games and he usually picks different activities from them, then I am there when he explains to others. So, he helps other children, even those who can’t; he tells them that yes, my name is, so he talks a lot about it. (Interviewee 1)

## Theme 2: helping parents promote children’s healthy lifestyles

### Motivating children who want to stay at home and play on screens

Parents commented on their difficulties keeping their children motivated when they wanted to stay home and play games on the TV or iPad. Parents saw their role as promoting a healthy lifestyle but found it challenging to address children’s interest in sedentary activities such as video games. For example, one parent mentioned, “He wants to stay and play [video games], but I don’t want to. He can’t just play until he goes to bed at half past seven. Sometimes he says, mum, I want to stay at home. I don’t know, it’s hard” (Interviewee 11). They also mentioned that “in autumn and winter, it’s just a lot of YouTube, and it doesn’t feel like there is not much learning going on” (Interviewee 8).

On the other hand, parents who successfully kept their children engaged in the activities recognised the benefits of PA for their children. One parent stated, “It’s such a positive impact. There’s no phone, no TV” (Interviewee 12). Some parents highlighted a notable change in their children after they started taking part in the activities: “Before the Open Activities, they just sat at home; it’s good that the children (now) are out and active” (Interviewee 2).

Many participants emphasised the importance of having something to do beyond using electronic devices such as iPads and mobile phones. During the observations, parents noted the lack of sports clubs and other activities in the area. They mentioned they would have to travel to different areas to participate in such activities. The parents also commented that there was nothing comparable to the Open Activities programme in the area.

The activities give the community and then the children something to do. It has become so much that they just sit in front of iPads and computers and mobiles and stuff like that, and I don’t like it so much, so I’m so happy that we now go to an activity, so we get something to do together (Interviewee 5).

### Transferring health-related learning from the activities to the home

One aspect of the activities that motivated the children to stay involved was the healthy habits quiz at the end of the sessions. During this time, the health coordinator gathers the children and presents a question with multiple options. One of the older children was asked to read the quiz aloud as a volunteer. It was observed that the children were very enthusiastic about discussing the different options and ‘guessing’ the correct answer to the quiz, and the volunteers were visibly very proud of their contribution. For example, the quizzes taught children the importance of eating vegetables or drinking water instead of soda. One parent commented, “When they come home and we sit and eat, it becomes like this; ‘where are the vegetables, where are they?’” (Interviewee 8). Parents have also noted that this moment keeps their family interested in being active and making changes at home to eat healthier and be more aware of the food they buy.

You learn a lot from each other as well as from these quizzes that are usually held at the end of each activity, about eating healthily, the way you live, that’s interesting, like how we should pay attention to things when we buy food (Interviewee 9).

## Theme 3: improving family dynamics through engagement in PA

### Creating memories by engaging in PA together

In addition to improving PA levels and healthy habits, parents felt that spending time together was an essential function of the activities. They recognised that the activities provided opportunities for meaningful memories and conversations with their children, strengthening their relationship with and support for each other. One parent noted that the activities have facilitated more open communication and allowed them to discuss situations that arose during the activities later.

She is happy when I am there, so we also talk about the activities that we have together. It’s great that we can reflect afterwards on what has gone well. Like, she might have been a little disappointed with her friend who didn’t behave as she should, so she can bring it up with me, and we can talk about it (Interviewee 9).

Parents reported that the activities enabled them to spend quality time with their children, which was often difficult due to their busy lifestyles. During the observations, it was noted that some parents had much fun, which impressed their children. As a result, they created everyday experiences by enjoying the same activities together.

You get memories, so when you leave, the children say, ‘Dad did this’, ‘It was fun or Yes, I see, but you do it like this, you felt’. We can talk about all of this. You’ve got a memory now together (Interviewee 12).

### Transforming parents from passive to active players

Several parents mentioned that they did not spend enough time playing with their children because they were not good at playing and lacked the motivation to play outdoors. For instance, one parent explained, “I think we parents are bad at playing. So, we play indoors, but outside, we become a little lazier” (Interviewee 10). Other parents explained that they wanted to play with their children before joining the activities but did not have enough time to find activities in their area as they were busy with work or household chores. A parent commented, “I don’t have that much time. They’re in school, and you’re tired in the evenings, so you don’t have as many activities together with your child” (Interviewee 9). Some parents described feeling disappointed when they could not meet their children’s play needs. The Open Activities allowed them to have fun with their children by ‘drop-in’ when possible.

On the weekends, for example, I have to cook and wash the clothes that will be used in the week. I have to prepare their [children’s] other clothes, so they create chaos in the home. It has happened several times that my son has come to me and said, ‘Dad, can we play football?’ But I’m so tired but he just wanted to play with me. He wants to have something to do with me and the Open Activities, it is so good that the children see how dad has fun and plays with them in several games, and is active with them (Interviewee 12).

Parents recognised that engaging in the activities changed their relationship with their children from passive to more active players.

### Removing the shame of not having the resources to engage in PA

Most parents mentioned financial and time resources as barriers to supporting their children’s regular involvement in PA. These barriers created tensions and feelings of guilt among parents, who were unable to cope with the planning and costs of certain activities. One parent described the Open Activities as “It makes a lot of difference. I mean, football costs 2000 SEK per term (approximately 180 EUR). Not everyone can afford it, or many people can’t afford it. So, these open activities are fantastic opportunities for movement and community” (Interviewee 10). In this way, the provision of organised activities by the Open Activities programme was described as alleviating parents’ feelings of guilt and shame.

You start to feel ashamed that you don’t have the finances or the strength to be able to give your children different types of activities and games, so if we didn’t have the Open Activities, honestly, it would take time to collect money. You have to collect money, you have to go to a place, and all this you have to plan, but when Open Activities organises them, it’s like a certain time the children can play with dad. It’s so valuable. (Interviewee 12)

Another concern raised by some parents was that although they might be able to raise money to support one of their children’s PA activities, most families in the area had several children. As a result, they may choose to avoid these activities altogether to avoid having to choose between their children.

What’s available in these areas costs money and not everyone can afford it and it’s expensive, yes, so sport is very expensive and it’s difficult because many people have many children and therefore it’s difficult to keep up with all of them so you avoid them, how should I do? I can’t pay for one and not let the others go so that they sit there at home for that reason (Interviewee 1).

## Discussion

This study aimed to explore parents’ experiences with the Open Activity programme in socioeconomically disadvantaged areas, focusing on family engagement in PA and perceived changes in family dynamics. The findings illustrate aspects of the programme that families perceived as key to facilitating children’s engagement in PA. These included creating a safe space and equal participation, supporting children using knowledge about healthy lifestyles at home, and fostering enjoyable involvement in PA. These aspects partly reflect what could motivate parents’ and children’s engagement in the programme in socially disadvantaged areas. They also highlight the complexity of health promotion interventions in these areas. Furthermore, the findings suggest that involving parents and children in PA helps parents become more aware of their children’s need for parental connection and being active.

### Creating a safe space and equal term participation

Engaging groups from socioeconomically disadvantaged neighbourhoods in family-based programmes can be challenging [37]. Negative experiences with professional agencies, fear of stigma, and language and cultural differences can contribute to this challenge [38]. The findings of this study suggest that providing activities tailored to children’s ages and needs allows children to feel safe trying new activities, which in turn maintains family engagement in the activities. The programme fostered positive experiences by ensuring the inclusion and equal participation of all the children, regardless of their needs, resources, language difficulties, or cultural preferences. This finding is consistent with previous research on inclusive outdoor play, which has shown that children perceive settings as inclusive when they provide equal opportunities for participation, a welcoming atmosphere, and a sense of involvement for all children [39, 40].

In this study, participants described equal terms as characterised by a perceived right to play and to be treated equally during play. This treatment includes being free to take on different roles during activities and receiving equal attention from staff [39]. For example, all children and parents were given equal attention by being asked by their names for their opinions, and all children were allowed to volunteer to read the quiz at the end of each session and ‘guess’ the correct answer. Such gestures of attention can help individuals feel comfortable taking interpersonal risks, such as talking to new peers or trying new group activities. This finding is consistent with the child development literature, which suggests that feeling psychologically safe is crucial for children to engage and develop [41].

Shy children may find it challenging to make friends in specific settings, such as school, where peers may not be as welcoming. Therefore, feeling comfortable and safe while playing can motivate them to continue participating [39]. Similarly, for parents of shy children, experiences that increase their children’s social contact are seen as incentives to continue participating in family-based PA programmes [42]. This study’s findings highlight how parents perceived their (shy) children to feel safe during activities, free from competition and judgment. As other research shows on PA in disadvantaged areas, families perceive programmes as inclusive and secure when they focus on cooperative games that foster inclusion and allow children to be themselves and fit in regardless of their sport or physical skills [43, 44].

Furthermore, the findings of this study suggest that a sense of belonging is also perceived by families, who describe positive group experiences with other parents and children. This group experience included respecting parents’ and children’s differences and supporting those who needed support; for example, if they had difficulties with the Swedish language, they were supported by using non-verbal communication to explain the activities. This group experience aligns with Jukes et al.’s [45] qualitative systematic review, which identified positive group experiences of egalitarianism and trust/confidentiality and tailored the programme to the needs of the children as critical facilitators for engaging families in PA programmes.

### Supporting children in transferring knowledge about healthy lifestyles back home

People living in socioeconomically disadvantaged areas are more likely to have lower levels of PA and to experience adverse health outcomes associated with an inactive lifestyle than their less disadvantaged peers are [46]. Previous studies have identified parental barriers to participation in family-based interventions, including competing demands associated with work commitments, busy schedules, and childcare responsibilities [42, 45]. In addition to these barriers, the participants in this study highlighted the challenge of competing with screen-based activities, such as playing video games on television, phones, or iPads, particularly during adverse weather conditions such as autumn and winter. This finding is consistent with research indicating that screen-based activities can reduce PA engagement among children in socioeconomically disadvantaged neighbourhoods [47, 48]. This reduction in PA levels is because time spent on screen-based activities may displace physical active time [49]. Lu et al. [50] reported that children from lower socioeconomic status (SES) families spent less time playing outdoors and more time on screens than children from higher SES families, resulting in socioeconomic disparities in outdoor play and screen-based activities. One possible reason for children’s time spent on screen-based activities is the limited availability of sports facilities or physical activity opportunities in disadvantaged areas [6, 51]. This lack of opportunities can leave children and young people with little or nothing to do in their spare time [47, 52]. Participants perceived the programme’s activities as providing accessible and health-promoting activities for the community and children.

The quizzes at the end of the activities were also noted to provide knowledge about healthy habits that children then transferred to their homes. For instance, parents indicated that quizzes encouraged their children to ask about vegetables while having dinner or drinking more water instead of soda. This finding is significant, as the ability to access, understand, and transfer knowledge about healthy lifestyles is lower in groups with low social status and financial deprivation [53]. A low ability to apply and transfer health-related knowledge is also associated with lower health outcomes, unhealthy behaviours, and higher healthcare utilisation rates [54].

### Fostering enjoyable participation in PA

Experiencing difficulties spending quality time with their children leads to parents feeling less motivated and tired or lazy to play outdoors with them. Research suggests that parents living in low SES areas have a reduced desire and motivation to engage in PA due to structural time constraints and parents’ work commitments [55]. Parents reported that during the Open activities programme, they felt closer to their child, had fun together, and communicated more openly about sensitive situations that arose during the activities. Child-parent bonding and the ability to set family time have been identified as facilitators of engagement in family-based programmes, suggesting the potential for participation in maintaining a healthy child-parent relationship during childhood and early adolescence [56]. This engagement is important because it indicates that co-participation in meaningful PA may lead to long-lasting changes in family dynamics that may be particularly beneficial later when adolescents’ PA levels begin to decline [4].

The participants reported dismissing or not responding to their children’s need for parental connection through play before participating in the programme. In the Open Activities programme, parents and their children are active together, playing the same games as peers rather than just monitoring or facilitating the activity. This engagement suggests that parent-child participation [56, 57] during the programme may have supported increased parental awareness. However, further research is needed to explore how involvement in PA together with their children can help parents’ awareness of their role as supporters or models of their children’s lifestyle. Higher parental education may be linked to increased parental participation in sports, greater involvement in leisure activities, and greater awareness of the health benefits of PA [58]. Improving parents’ awareness of PA can be a critical initial step for interventions aimed at increasing children’s PA and promoting more PA among parents themselves. If parents recognise the advantages of PA for themselves, they are more likely to encourage their children to engage in it [14]. Future family-based PA programmes could consider raising parents’ awareness of the benefits of spending enjoyable PA time with their children to maintain a healthy lifestyle, including physical activity and a healthy parent-child relationship [58].

### Strengths and limitations of the study

The relatively small number of participants is a potential limitation of the study. However, reaching socially disadvantaged groups has proven challenging due to mistrust of research or researchers, fear of authority, and the perception that participation will not benefit them [59]. These challenges have resulted in low participation rates or the exclusion of these groups from public health research [59]. The researchers’ long-term involvement in the program allowed them to build trust with a few participants, who then agreed to participate in the interviews. Perhaps participants who were dissatisfied with the activities withdrew from the program before the researchers participated in the sessions. This withdrawal may explain why negative or alternative experiences are lacking in the descriptions provided by the participants. Another potential limitation is that all the researchers were female, which could have potentially hindered the recruitment of more fathers to the study due to cultural or religious values. However, spending time and building trusting relationships with the participants facilitated the collection of reliable accounts and rich observations. A strength of this study lies in the triangulation of different modes of data collection, such as interviews and participant observations. The involvement of three researchers (LF, JG, and SA) in data collection and transcript analysis enhanced researcher peer debriefing, reflexivity, and triangulation [60]. The participation of two researchers (M-L H and GN), who were not involved in data collection, in the data analysis to review themes was influential in establishing team consensus and confirmability [60].

## Conclusion

Families living in socioeconomically disadvantaged areas face multiple challenges to participation in PA programmes [37]. The Open Activities programme provided free drop-in and outdoor activities to support families’ engagement in PA. The findings of this study suggest that a perceived equal treatment and safe space motivated children, including shy children and those with different support needs, to participate in PA. Children’s increased knowledge of healthy lifestyles was supported by quizzes, and parents’ awareness of the benefits of PA was supported by spending quality time with their children during the activities. Further research is needed to explore how to facilitate parental awareness of children’s PA in socioeconomically disadvantaged areas to maintain healthy habits and child-parent relationships.

## Electronic supplementary material

Below is the link to the electronic supplementary material.

Supplementary Material 1

## Data Availability

No datasets were generated or analysed during the current study.
