# Supplementary material for: The open health-promoting activities programme: redefining health promotion and family dynamics by engaging parents in socioeconomically deprived Swedish communities
Source: BMC Public Health. 2025 Feb 12;25:580. doi: 10.1186/s12889-025-21799-0 (PMC11816766; doi:10.1186/s12889-025-21799-0)
Supplement: Supplementary file 1 — Supplementary Material 1 [file 12889_2025_21799_MOESM1_ESM.docx]

**Supplementary 1 - Interview guide**

**Previous experiences of outdoor Physical Activity**

1. What were your experiences with outdoor physical activities (outdoor or club activities) before you started participating in the Open Activities?

**Experience in participating in the Open Activities**

2. How did you come into contact with the Open Activities programme?

- Did you know any of the families or leaders before participating?

3. What were your expectations/hopes of Open Activities before you started? What were they? Did you have any fears, worries, concerns, which ones?

4. Can you tell us about your family's participation in Open Activities?

- Who participates? and how long have you been involved? If only one child participates but has several siblings, why don't the others participate? If only one parent participates, why doesn't the other participate?
- Considering that you and your child(ren) participate on Saturdays - how did you experience it on your weekend/weekday?

5. How would you describe Open Activities?

- Which activities do you like/appreciate, and what do you appreciate about these activities?
- Is there anything you do not like/appreciate?
- Can you describe a situation/activity that was experienced well (fun/engaging) and one that was less good during open activities?

**Open Activities’ potential impact on the personal and family well-being of the participant.**

6. What has it meant for your family to participate in Open Activities?

**Facilitating and hindering factors for participation**

7. What is it that makes you continue/not able to continue participating in open activities?

8. What do you see as the main barriers for your children to participate in outdoor physical activities?

9. What do you think about the activities taking place outdoors and being drop-in, i.e. that they do not require registration?

10. Do you have anything else you want to say in relation to what we have discussed? Thank you for your participation!
